# Evaluation of an anti-stigma campaign related to common mental disorders in rural India: a mixed methods approach

**DOI:** 10.1017/S0033291716002804

**Published:** 2016-11-02

**Authors:** P. K. Maulik, S. Devarapalli, S. Kallakuri, A. Tewari, S. Chilappagari, M. Koschorke, G. Thornicroft

**Affiliations:** 1 Research & Development, George Institute for Global Health, New Delhi, India; 2 George Institute for Global Health, University of Oxford, Oxford, UK; 3 Centre for Global Mental Health, Institute of Psychiatry, Psychology and Neuroscience, King's College, London, UK

**Keywords:** Common mental disorders, community-based, India, low- and middle-income countries, mental health awareness, stigma.

## Abstract

**Background:**

Stigma related to mental health is a major barrier to help-seeking resulting in a large treatment gap in low- and middle-income countries (LMIC). This study assessed changes in knowledge, attitude and behaviour, and stigma related to help-seeking among participants exposed to an anti-stigma campaign.

**Method:**

The campaign, using multi-media interventions, was part of the SMART Mental Health Project, conducted for 3 months, across 42 villages in rural Andhra Pradesh, in South India. Mixed-methods evaluation was conducted in two villages using a pre-post design.

**Results:**

A total of 1576 and 2100 participants were interviewed, at pre- and post-intervention phases of the campaign. Knowledge was not increased. Attitudes and behaviours improved significantly (*p* < 0.01). Stigma related to help-seeking reduced significantly (*p* < 0.05). Social contact and drama were the most beneficial interventions identified during qualitative interviews.

**Conclusion:**

The results showed that the campaign was beneficial and led to improvement of attitude and behaviours related to mental health and reduction in stigma related to help-seeking. Social contact was the most effective intervention. The study had implications for future research in LMIC.

## Introduction

Stigma is an attribute, behaviour, or reputation which is socially discrediting in a particular way: it causes an individual to be mentally classified by others in an undesirable, rejected stereotype rather than in an accepted, normal one (Goffman, 1963). Stigma has also been conceptualized as a problem with three elements: knowledge (ignorance/misinformation); a problem of attitudes (prejudice); and a problem of behaviour (discrimination) (Thornicroft *et al.* 2007). Corrigan *et al.* (2012) outlined three strategies for addressing stigma that can be understood from the theoretical perspectives of knowledge, attitude and behaviour – educational materials that target inaccurate knowledge and stereotypes and try to change them; interpersonal contact with members of a stigmatized community that helps to reduce prejudice and change behaviour; and public protests against those who stigmatize other groups such that there is a behaviour change. Two reviews (Corrigan *et al.* 2012; Thornicroft *et al.* 2016) have shown that interpersonal contact and to a lesser degree educational materials are intervention strategies that have been effective to some degree.

Stigma related to mental illness is a widespread issue in the world (WHO, 2001), and is a major impediment towards help-seeking for mental disorders (Clement *et al.* 2015). Especially in low- and middle-income countries (LMIC), lack of awareness about mental health, especially common mental disorders (CMD) such as depression, anxiety, suicidal risk and emotional stress, and stigma against using mental health services are major barriers against help-seeking. This is reflected in estimates that only 15–25% people suffering from mental disorders receive any kind of treatment in LMIC (WHO World Mental Health Survey Consortium, 2004). In spite of that, little data exist about suitable interventions in LMIC that address stigma (Semrau *et al.* 2015).

This paper reports the mixed-methods evaluation of an anti-stigma campaign based on the principles outlined by earlier research (Corrigan *et al.* 2012; Thornicroft *et al.* 2016), conducted in rural India that focused on increasing mental health knowledge and awareness. The main objective was to identify any changes in mental health knowledge, attitude and behaviour and stigma related to help-seeking between pre- and post-intervention This campaign was part of a larger study designated SMART (Systematic Medical Appraisal, Referral and Treatment) Mental Health, involving task shifting, and using innovative mobile-based strategies for mental health services delivery for managing CMD, by primary-care health workers in rural India (Maulik *et al.* 2015).

## Method

### Study site

SMART Mental Health was conducted in West Godavari district in the south Indian state of Andhra Pradesh. The anti-stigma campaign was implemented across 42 villages (30 of which belonged to Scheduled Tribe (ST) area and was supported by a small grant, and 12 to non-Scheduled Tribe areas supported by a larger grant). Both these sets of villages were from the same district and the villagers spoke Telugu. The health systems are similar, though services are scarcer in ST areas. The ST villages are more remote (http://aptribes.gov.in/statistics.htm), smaller in size, and have poorer health indicators (Ministry of Tribal affairs, 2014). It was predetermined at the protocol development stage that the formal evaluation would be conducted in only two villages out of the 12 villages in the non-ST areas. The study in the non-ST villages had been supported for a longer period of time and had a larger budget. Both of these factors provided an opportunity to formally evaluate the anti-stigma campaign in that area.

The two villages (eligible adult population of 2764) were selected purposively and the criteria used for selection were: distance of each village <40 km from the field office; eligible population in each village is of average size (~1500); each village has at least two village health workers (Accredited Social Health Activists; ASHAs); and each village is under a different primary health centre.

### Study population

Evaluation was made on all eligible adults aged ⩾18 years who provided consent and were available for interview. Those who were too sick or were not able to comprehend the questions due to severe physical or mental illness were excluded.

### Study design

The evaluation of the anti-stigma campaign involved a pre-post study design, using a mixed-methods approach. No control group was present. Pre-intervention data were collected in March 2015. The intervention (the anti-stigma campaign) was delivered over a 3-month period from the middle of March until the end of June 2015. The post-intervention data including qualitative data were collected after the end of the intervention in June–July 2015. While all post-intervention quantitative data were collected then, data on the Barriers to Access to Care Evaluation (BACE; Institute of Psychiatry, King's College London, 2011) were re-collected for the whole population in October as there was an error in the programming of the software which resulted in the questionnaire being skipped for many individuals.

Data are reported as per STROBE (Strengthening the Reporting of Observational Studies in Epidemiology) guidelines for reporting observational studies (von Elm *et al.* 2007).

### Development of the intervention

Prior interventions used in stigma research or programmes for mental health and other health conditions such as HIV were identified, with a focus on India or other LMIC. Discussions with experts in the field (G.T. and M.K.) helped identify key strategies and programmes relevant to our study. The strategies identified were:

(1) *Developing printed information, education and communication (IEC) materials*. This strategy involved developing brochures, pamphlets and posters on signs and symptoms of CMD such as depression, suicidal risk, stress and how they differed from severe mental disorders; the need for seeking treatment and how it could impact health; issues of stigma related to mental health prevalent in the community. Vignettes on CMD were included in the brochures as examples and discussed. The information from earlier research was adapted to local needs by conducting formative research (Maulik *et al*. 2016), and the documents were translated into Telugu. CMD were described and the community understood depression/stress/anxiety and suicidal risk. Local terms to describe stress were incorporated in the materials. The brochures and pamphlets were used in the door-to-door campaign and community meetings to raise mental health awareness and discuss issues related to stigma. This was repeated 3–4 times with each household in the villages during the intervention phase. The posters and pamphlets were shared with local government offices, schools, and primary health centres and were displayed on their walls or notice boards. This strategy addressed misinformation (lack of knowledge) and used education-based strategies to address that issue.
(2) *Involving a person with CMD to talk about his experience*. We identified one person and his caregiver who were willing to have a video made of their experience for sharing with others. This video was screened and discussed during the campaign. This used a social contact strategy to raise awareness and reduce stigma.
(3) *Developing a promotional video on mental health, stigma and the SMART Mental Health project*. A local film actor promoted the video and spoke about CMD, and this was screened during the campaign. This used an education strategy to raise awareness.
(4) *Staging a drama by a local theatre group*. A theatre group was identified who already had a script on domestic violence, depression and the need for getting treated. The script was modified to complement the information in the IEC materials. Live performances of the drama were organized in eight villages, including the two where the evaluation was conducted. Additionally, video recordings of it were shown to those who missed the live performances, or in other villages where the live performance was not possible. Short clippings were also shown during the door-to-door campaigns. This used an education-based strategy to increase knowledge and attitudes related to CMD.

### Mixed-methods evaluation

Quantitative data were collected at the pre- and post-intervention phases. This was conducted by trained field interviewers using a tablet. The interviewers made it clear at the start of the interview that the intervention and the assessments were all related to CMD. Besides questions on socio-demographic status and health-related topics, the key instruments that were used for measuring stigma and mental health awareness were:

• *Barriers to Access to Care Evaluation: Treatment Stigma Subscale (BACE-TS version 3) (Institute of Psychiatry, King's College London,* *2011*). This is a 12-item questionnaire with a 4-point Likert scale asking questions relevant to stigma associated with seeking care for mental illnesses. BACE has been found to have moderate to good reliability and good construct validity (Clement *et al.* 2012). The questionnaire was translated into Telugu and back-translated, but no differences were identified. Test–retest reliability, assessed using a standardized Cronbach's alpha test, was 0.85 indicating good internal consistency.
• *Mental Health Knowledge, Attitude and Behaviour (KAB;* Lund *et al*. 2012). This is a 16-item questionnaire developed for the PRIME study (Lund *et al*. 2012) and is based on a number of other tools. It uses a 5-point Likert scale that ascertains mental health knowledge, attitude and behaviours as per the framework for understanding stigma suggested by others (Thornicroft *et al.* 2007). This was translated into Telugu, and back-translated into English. The subgroups were identified based on discussion with experts and were not based on any psychometric analyses, hence did not have the properties of a scale.

Qualitative data were collected from community members, ASHAs, village leaders and field interviewers, using focus group discussions (FGDs) and in-depth interviews (IDIs). They were conducted after the intervention using a set of open-ended questions that explored their views about stigma against mental health and help-seeking for mental disorders. They were also asked to provide their perceptions on positive/negative attributes of each strategy used in the campaign. Opinions were also sought about marrying/living/working with people with CMD; knowledge about CMD and the need to seek treatment; and societal prejudices. The interviews were conducted in the local language (Telugu) and was audio-recorded. Qualitative data were collected by trained researchers. The recordings were transcribed and translated to English.

### Data management and analysis

Quantitative data were stored in a secure server based at the George Institute India. A statistical plan was developed prior to analysing the data, and drew on analyses done in earlier research (Clement *et al.* 2012). Response frequency and mean scores were derived wherever applicable for the socio-demographic variables and individual questions of the two questionnaires. The difference in mean score for the KAB items were calculated. The mean scores for each item on the BACE; proportion of respondents identifying each item as a possible barrier (‘a little’ or more); proportion of respondents identifying each item as a major barrier (‘a lot’); and the rank of each item as a barrier based on the proportion who identified it as a major barrier have been estimated. The mean scores and rank of different items between the pre- and post-evaluation phases were compared using paired *t* tests and McNemar's χ^2^ test using SAS v. 9.04 (SAS, 2016). These were performed only for subjects for whom information was available at both time-points – pre- and post-intervention stages, hence there were fewer observations compared to the total number of observations at each stage.

Qualitative analyses was based on grounded theory and used a thematic framework approach to identify common emerging themes. First, each audiotaped, IDI/FGD was transcribed verbatim, and the textual data files were imported into Nvivo 9 (NVivo, 2010). The researchers (A.T., S.K., S.D.) initially familiarized themselves with the data and during that process identified broad thematic areas. A coding scheme was formulated using an inductive approach. All transcripts were reviewed to identify recurrent themes across individuals and groups, which were then refined into codes. Two researchers (S.K. and A.T.), working together, defined each code category and then individually proceeded to code the text of the interviews. Discrepancies in coding were identified, and consensus was obtained through discussion and clarification of coding categories. Results obtained from the quantitative and qualitative research were collated, using a concurrent triangulation of data from both methods to derive a comprehensive understanding of the anti-stigma campaign (Hanson *et al.* 2005).

## Ethical standards

Ethics approval for the study was obtained from the Independent Ethics Committee of the Centre for Chronic Disease Control, New Delhi. Written informed consent was obtained from all participants. The authors assert that all procedures contributing to this work comply with the ethical standards of the relevant national and institutional committees on human experimentation and with the Helsinki Declaration of 1975, as revised in 2008.

## Results

The two villages had a similar sociodemographic profile as the larger set of 12 non-ST villages with respect to mean age (~40 years), gender distribution (~60% female), education (~30% with no schooling), marital status (~80% married), occupation (~35% being housewife/retired) (data not shown). Out of 4600 people in the two villages, 2764 (60.1%) eligible adults were identified. The pre- and post-intervention data were collected from 1576 (57%) and 2100 (76%) of the eligible adults, respectively. Due to the presence of local industries which are seasonal, a large proportion of the villagers had left during the pre-intervention phase to work there, but returned later on. While we cannot comment on when they returned and for how long they received the intervention, the pre-post assessment is based on only paired observations who were interviewed at both times. As discussed in the study design the BACE was re-administered, and 1783 out of the 2100 interviewed at post-intervention could be interviewed (Supplementary Table S1).

### Quantitative analysis

The socio-demographic characteristics of the study participants at both pre- and post-intervention phases were similar with more than 55% being women; about half being employed in unorganized sectors involving farming, contract labour, and small shops; about a third having no formal education; and more than 80% being ‘currently married’. The mean age of the participants was around 42 years at both time-points (Table 1).

**Table 1.** Sociodemographic characteristics of the study participants

Characteristic	Pre-intervention (*N* = 1576), *n* (%)	Post-intervention (*N* = 2100), *n* (%)
Gender		
Female	929 (58.95)	1150 (54.76)
Male	647 (41.05)	950 (45.24)
Occupation		
House wife/retired	612 (38.83)	760 (36.19)
Organized sector	40 (2.54)	59 (2.81)
Unorganized sector	785 (49.81)	1090 (51.90)
Other	139 (8.82)	191 (9.10)
Education		
Graduate/postgraduate	49 (3.11)	80 (3.81)
High school	267 (16.94)	422 (20.10)
Primary school	746 (47.34)	934 (44.48)
No school	507 (32.17)	640 (30.48)
Other	7 (0.44)	24 (1.14)
Marital status		
Currently married	1261 (80.01)	1703 (81.10)
Never married	151 (9.58)	199 (9.48)
Separated/divorced/widowed	164 (10.41)	198 (9.43)
Age (years)		
Mean (s.d.)	42.8 (15.79)	41.8 (15.65)
Range	18–90	18–90

*N*, Total number of participants in each phase.

*n*, Number of participants with particular characteristic.

Television was the commonest source of information on mental health, and hospitals and clinics were identified as the places to receive treatment for mental disorders. About a third knew of someone with a mental illness. Within the *knowledge* domain majority felt that people with mental illness ‘tend to be violent’ and cannot lead a ‘rewarding life’, but they can be treated especially with medications. The *attitude* towards people with mental illness was ambivalent in that on one hand the majority felt that people with mental illness ‘should not get married’ and ‘should not be given any responsibility’, but on the other the majority felt that people with mental illness are ‘far less of a danger than supposed’ and that society needed to have a ‘tolerant attitude towards people with mental illness’. From a *behaviour* perspective, the majority were willing to share their life with someone with mental illness either at work, or being in a relationship or having someone with mental illness as neighbours. The majority were willing to share personal mental illness details with family (Supplementary Table S2).

Compared to the pre-intervention data, the post-intervention data showed statistically significant improvement of scores (lower scores) on most of the *attitude* and *behaviour* domain-related questions except the item ‘*mentally ill people shouldn't get married’*, which showed a statistically significant increased score by 0.13 (s.d. = 1.89) (*p* = 0.01). No significant *knowledge* gain was observed. By contrast, there was a statistically significant worse score on the item  ‘*people with mental illness cannot live a good, rewarding life*’ (Table 2).

**Table 2.** Change in Knowledge, Attitude and Behaviour scores between pre- and post-intervention

Domain	Question	Pre-intervention mean (s.d.)	Post-intervention mean (s.d.)	Difference in mean (s.d.), *n*	*p* value
Knowledge	Mentally ill people tend to be violent	2.2 (1.21)	2.2 (1.27)	−0.01 (1.76), 1183	0.8428
	People with mental illness cannot live a good, rewarding life	2.1 (1.05)	1.7 (1.00)	−0.30 (1.48), 1238	<0.0001
	People with severe mental health problems can fully recover	1.7 (0.92)	1.7 (0.96)	−0.05 (1.29), 1352	0.1226
	Medication can be an effective treatment for people with mental health problems	1.6 (0.89)	1.5 (0.88)	−0.03 (1.22), 1396	0.2927
Attitude	Mentally ill people shouldn't get married	2.3 (1.31)	2.4 (1.43)	0.13 (1.89), 1252	0.0137
	People with mental health problems are far less of a danger than most people suppose	2.0 (1.01)	1.6 (0.85)	−0.34 (1.29), 1269	<0.0001
	We need to adopt a far more tolerant attitude toward people with mental illness in our society	1.5 (0.80)	1.2 (0.56)	−0.23 (0.99), 1435	<0.0001
	People with mental health problems should not be given any responsibility	2.2 (1.23)	1.9 (1.20)	−0.31 (1.68), 1335	<0.0001
Behaviour	If you suffered from a mental health problem would you tell your family or friends^a^	2.4 (0.88)	2.8 (0.49)	0.42 (0.98), 1575	<0.0001
	I would be willing to live with someone with a mental health problem	1.9 (1.07)	1.6 (1.03)	−0.27 (1.44), 1406	<0.0001
	I would be willing to work with someone with a mental health problem	1.9 (1.11)	1.6 (1.01)	−0.29 (1.39), 1404	<0.0001
	I would be willing to live nearby someone with a mental health problem	1.9 (1.10)	1.6 (0.98)	−0.31 (1.41), 1393	<0.0001
	I would be willing to continue a relationship with a friend who developed a mental health problem	1.8 (1.01)	1.5 (0.90)	−0.26 (1.28), 1412	<0.0001

*p* value is calculated using paired *t* test; *n*, participants who responded to each item at both times.

a Coded differently – no one = 1, friend = 2, family = 3 (higher scores indicating bias towards family).

The BACE responses indicated a low level of stigma and this reduced even further following the intervention. The major difference observed between pre- and post-intervention was that the proportion of people who had identified each item as even a possible barrier or had identified them as a major barrier, had both reduced. The only two items which continued to rank among the top three major barriers at both time points were ‘*concern that my children may be taken into care or that I may lose access or custody without my agreement’* and ‘*concern about what people at work might think, say or do’* (Supplementary Table S3–5).

All the items on the BACE showed statistically significant lower scores at post-intervention compared to pre-intervention and this was true for the total mean BACE score also (Table 3).

**Table 3.** Change in mean scores for each barrier in the Barriers to Access to Care Evaluation (BACE) – Treatment Stigma Subscale

Question	Pre-intervention Mean (s.d.)	Post-intervention Mean (s.d.)	Difference of mean (s.d.), *n*	*p* value
Concern that I might be seen as weak for having a mental health problem	0.4 (0.67)	0.1 (0.39)	−0.23 (0.772), 1348	<0.0001
Concern that it might harm my chances when applying for jobs	0.6 (0.81)	0.1 (0.34)	−0.36 (0.827), 160	<0.0001
Concern about what my family might think, say, do or feel	0.4 (0.67)	0.2 (0.45)	−0.24 (0.808), 1348	<0.0001
Feeing embarrassed or ashamed	0.4 (0.66)	0.2 (0.40)	−0.18 (0.759), 1348	<0.0001
Concern that I might be seen as crazy	0.4 (0.69)	0.1 (0.37)	−0.24 (0.775), 1348	<0.0001
Concern that I might be seen as a bad parent	0.4 (0.67)	0.2 (0.38)	−0.23 (0.756), 1250	<0.0001
Concern that people I know might find out	0.4 (0.67)	0.1 (0.35)	−0.26 (0.708), 1348	<0.0001
Concern that people might not take me seriously if they found out I was having professional care	0.4 (0.66)	0.1 (0.43)	−0.28 (0.763), 1348	<0.0001
Not wanting a mental health problem to be on my medical records	0.3 (0.75)	0.1 (0.23)	−0.28 (0.791), 1348	<0.0001
Concern that my children may be taken into care or that I may lose access or custody without my agreement	0.4 (0.73)	0.2 (0.40)	−0.22 (0.842), 1244	<0.0001
Concern about what my friends might think, say or do	0.4 (0.70)	0.1 (0.37)	−0.30 (0.784), 1348	<0.0001
Concern about what people at work might think, say or do	0.5 (0.73)	0.2 (0.52)	−0.24 (0.883), 1348	<0.0001
Overall mean	0.4 (0.08)	0.1 (0.04)	0.3 (0.09)	<0.0001

*n*, Number of participants at both pre- and post-intervention.

*p* value is calculated using paired *t* test.

Table 4 shows that for each question on the BACE, the proportion of participants who found that each barrier was a major issue (‘a lot’) had reduced significantly between pre- and post-intervention.

**Table 4.** Change in proportion between pre- and post-intervention on Barriers to Access to Care Evaluation (BACE) – Treatment Stigma Subscale who found barriers affecting them ‘a lot’

Question	*n*	% Reporting as major barrier (a lot) pre-intervention	% Reporting as major barrier (a lot) post-intervention	*p* value
Concern that I might be seen as weak for having a mental health problem	1348	1.78	0.14	<0.0001
Concern that it might harm my chances when applying for jobs	160	0.63	0.05	0.0196
Concern about what my family might think, say, do or feel	1348	1.52	0.05	<0.0001
Feeing embarrassed or ashamed	1348	1.52	0.05	<0.0001
Concern that I might be seen as crazy	1348	1.46	0.14	0.0003
Concern that I might be seen as a bad parent	1250	1.27	0.05	0.0013
Concern that people I know might find out	1348	1.33	0.05	0.0008
Concern that people might not take me seriously if they found out I was having professional care	1348	1.21	0.05	0.0008
Not wanting a mental health problem to be on my medical records	1348	4.70	0.05	<0.0001
Concern that my children may be taken into care or that I may lose access or custody without my agreement	1244	3.17	0.10	<0.0001
Concern about what my friends might think, say or do	1348	1.65	0.05	<0.0001
Concern about what people at work might think, say or do	1348	2.16	0.67	0.0112

*p* value is calculated using McNemar's χ^2^ test.

### Qualitative analyses

Overall, five FGDs and six IDIs were conducted in two villages:

• Four FGDs were conducted with community members (18 male, 15 female, age 22–65 years), two FGDs in each village segregated by gender.
• One FGD was conducted with field investigators (six male, four female, age 22–34 years).
• Four IDI were conducted with ASHAs (females aged 31–42 years), and two with a village leader from each village (a 54-year-old male and a 48-year-old female).

The results from all the FGDs and IDIs are collated and presented under five major themes (Table 5).

**Table 5.** Summary of qualitative research

Anti-stigma campaign strategies	Purpose	Themes	Findings	Examples of verbatim quotes
• IEC Material – brochure, pamphlets and posters • Video film of a person with mental disorder • Street play on domestic violence and mental disorder	• Explore knowledge about the process of delivering the anti-stigma campaign – appropriateness and usefulness of the campaign; relevance to local culture and settings • Assess the impact of anti-stigma campaign on the community environment	Awareness about the anti-stigma campaign activities	• Majority were aware of door-to-door campaign, drama and the video films	• ‘Programme was useful’…‘We understood that how people with psychological problem suffer and how it leads to depression’ (51-year-old female community member)
		Effective strategies used to create awareness	• Street play and video film were favourite medium in dissemination of information related to mental disorder	• An adult leader shared, ‘Out of all, how a girl suffers and how to [one] looks after that girl, and the whole characterization of that girl was very nice. The character initiated us to think about something’ (54-year-old male village leader) • ‘… we came to know about [Kiran] who was in hospital suffering from mental disorder and was cured by taking treatment’ (45-year-old female community member)
		Changes in knowledge about common mental disorders and stigma	• Gained knowledge about mental disorder and related issues • Increase in awareness on existing treatment facilities	• A leader mentioned: ‘Earlier we thought that mental illness can't be cured. Now we tell our family members and others that we should support the person facing such problem and take him/her to the doctor’ (48-year-old female village leader). • ‘… I hope that it may [be] cure[d] …’ while being married (Group's view)
		Access to treatment	• Less accessibility to healthcare facility for mental health treatment in the same village	• ‘We are taking medicine and treatment for this [mental illness] in other city, which is very far’ (52-year-old male community member)

#### Theme I. Awareness about the anti-stigma campaign activities

The majority of the participants were aware of the campaign activities, such as door-to-door campaign, drama and the video films, but few were aware about the posters and pamphlets exhibited in public places and primary health centres. All stakeholders felt that such interactive campaigns should be organized regularly.

#### Theme II. Effective strategies used to create awareness

Overall, most of the community members felt that they gained knowledge about CMD and related issues through the campaign. Most participants opined that the drama and the film of the person discussing his personal mental illness were the most effective strategies because they showed how people with mental illness suffer, and how it leads to depression. Some of the participants could also relate to the characters shown in the drama. Many participants mentioned that the drama and videos made them realize that they should not desert or abuse persons suffering from psychological problem, rather provide support to them.

#### Theme III. Changes in knowledge about CMD and stigma

During the discussions most of the participants cited different reasons for the cause of mental disorder. However, they indicated that the new knowledge resulted in a change in their perceptions about mental disorders and attitude towards people with mental disorders, e.g. they felt that marriage with someone having mental illness is not an issue for them anymore.

The village leaders agreed that increase in mental disorders was a cause of concern for them and they supported this campaign. They felt that the campaign was able to dispel some myths regarding mental illness.

Community members added that they felt confident approaching a person and persuading him/her or family members to seek treatment from a doctor.

#### Theme IV. Access to treatment

Some of the community members shared that they were not aware about existing treatment facilities before the implementation of this programme. They were concerned about the non-availability of treatment facilities in their villages and that they had to travel far to receive treatment.

#### Theme V. Suggested strategies

Almost all the participants wanted more plays and films on people with mental disorders for creating awareness about mental health issues. Some of the participants suggested organizing camps in their villages where they could interact with experts, or a doctor to clarify doubts. Some of the field investigators and ASHAs suggested using media to create awareness. The field investigators suggested involving school teachers such that mental health awareness can be imparted to school children.

## Discussion

This study used a mixed-methods approach to evaluate an anti-stigma campaign related to CMD using a pre-post design. To the best of our knowledge this is the first study from a LMIC that reports the results of such a campaign on a large community-based population. The results suggest that the knowledge of the study participants about mental health did not differ significantly following the intervention, but both their attitudes and behaviours did change for the better in most situations. This is one of the few studies globally, which measured changes in behaviour using a set of questions around hypothetical scenarios, although not in real-life situations. Stigma towards accessing mental healthcare was also reduced following the intervention.

The study design is limited by being a pre-post method, hence efficacy of the intervention could not be ascertained as in a randomized controlled study. Moreover, since it does not have a control group, the results need to be interpreted with caution. The KAB and BACE have not undergone stringent psychometric assessment within the specific study population, but nonetheless, both tools have undergone translation and back-translation and the test–retest reliability of the BACE was found to be good. Although the strategies used are generalizable to similar rural settings, the content and language may not be generalizable to other populations. All eligible adults in the villages were recruited, and the profile of the pre- and post-intervention population were similar, hence recruitment bias is negligible, even though this was not a random sample. The responses to the BACE at post-intervention were collected later and it may have resulted in attenuated effect. However, given the magnitude of change in BACE scores and the sample size, a less attenuated result would only increase the difference in scores and not have any significant impact on the implications.

### Changes in outcomes related to knowledge, attitude and behaviour, and help-seeking

It was understandable that most people received information about mental health via films and television shows, as these are the commonest media they are exposed to. Most participants identified hospitals and clinics as the primary areas for receiving care for mental illness, as generally people are mainly aware about severe mental disorders for which care was sought in hospitals and clinics. CMD were neither known as mental health conditions nor was treatment sought. However, about 5% of the participants preferred religious leaders/traditional healers, and earlier research has identified using similar services, too, in India (Hashimoto *et al.* 2015).

Comparing pre-post data on knowledge, attitude and behaviour, it was evident that significant changes were observed for attitude and behavioural components, with little impact on increase in knowledge. This has also been reported in a recent review which found similar results when exploring evidence-based interventions for reducing stigma and discrimination in mental health (Thornicroft *et al.* 2016). The results also indicate that at post-intervention more people agreed with the comment that ‘*people with mental illness cannot lead a good rewarding life*’. This contrasts with the other more positive views observed following the intervention. One reason for this could be that this particular question was interpreted in light of the quality of life. So while people felt that treatment helps and the mental health condition can improve, they were not sure of the overall impact on the quality of life and productivity. This needs further research. Mental health attitudes has been found to improve following anti-stigma and mental health awareness campaigns, but not knowledge, even in high-income countries, and no conclusive data are available for behaviour change. Data from LMIC are almost negligible and what little exists is inconclusive about the effectiveness of the interventions or the overall outcomes (Semrau *et al.* 2015).

Although in our study, the quantitative data failed to show significant knowledge gain at post intervention, qualitative data suggests changes in knowledge both among the community and key stakeholders, such as village leaders. However, while the quantitative assessment showed a small but statistically significant increase in people endorsing the statement that ‘*people with mental illness shouldn't get married*’, the qualitative data suggested that people's views varied. The reason for this is that while the overall attitude is that people with mental illness should not marry as that was thought to affect the spouses’ life, in the FGDs they opined that they did not see any harm in marrying someone with mental illness as it probably would get cured. This change in attitude could be because they had additional information about benefits of treatment for CMD through the anti-stigma campaign. Prior to the qualitative interviews the context of the campaign were re-emphasized and this may have helped them understand the point better.

Overall, stigma against help-seeking was low and reflects trends obtained from other research from LMIC (Semrau *et al.* 2015). A third of the population had a neighbour or family member with mental illness and that may have acted as an interpersonal contact resulting in reduced stigma. Little prior experience with mental health services may have also contributed to low scores on the BACE due to poor understanding of the stigma associated with seeking treatment. The anti-stigma campaign reduced stigma even further. Two concerns that continued to be ranked highly at post-intervention were concerns that the children may be taken away without agreement and what colleagues at the workplace may think of a person seeking mental health treatment – barriers identified in an earlier review of both quantitative and qualitative studies (Thornicroft, 2008; Clement *et al.* 2015). Both these issues have policy implications for confidentiality of personal health records at the workplace and policies around childcare and social support for parents with mental illness.

### Intervention strategies

This anti-stigma campaign used a number of intervention strategies and it is not possible to delineate which particular strategy was most effective. However, social contact, even if indirect in the form of a video showing a person with mental illness talking about his experiences, and the drama were clearly identified as the most effective strategies in the qualitative analyses. Social contact has been identified as an effective strategy in earlier research too (Corrigan *et al.* 2012; Thornicroft *et al.* 2016). Overall, the community found the anti-stigma campaign beneficial and wanted teachers and doctors to be involved too. They suggested using multi-media approaches, and organizing the campaigns via smaller camps. While in our study, no one commented about the benefits of the education material, it was apparent during the discussions that they had gained some information about treatment options and stigma from the mental health awareness materials shared with them. Mental health education has been found to be effective, especially in interventions conducted for more than 4 weeks (Thornicroft *et al.* 2016).

### Implications for future programmes and research

Addressing mental health stigma is essential to reduce treatment gap, as it helps to increase help-seeking. Developing strategies to reduce stigma and promote mental health are identified as key strategies in the World Health Organization's Mental Health Action Plan for 2013–2020 (WHO, 2013), and more randomized controlled trials are needed in Indian populations to generate evidence. Future research needs to explore newer techniques of sharing information related to stigma and discrimination, and develop strategies for specific populations such as women, students, and caregivers, as studies indicate that there are differences in the effectiveness of different strategies among population subgroups (Corrigan *et al.* 2012; Thornicroft *et al.* 2016). Research using mixed methods also needs to explore the level of stigma in the community and see if similar low levels are obtained and ascertain explanations for such. Strategies also need to be more inclusive and involve peer-led participatory models to encourage wider dissemination, especially for specific groups such as adolescents and women, who may have specific issues that lead to stigma and such could be identified and discussed more effectively through peer-led processes (Bulanda *et al.* 2014).

## Supplementary material

For supplementary material accompanying this paper visit http://dx.doi.org/10.1017/S0033291716002804. click here to view supplementary material
