# Supplementary material for: Evaluation of an anti-stigma campaign related to common mental disorders in rural India: a mixed methods approach
Source: Psychol Med. 2016 Nov 2;47(3):565–75. doi: 10.1017/S0033291716002804 (PMC5244444; doi:10.1017/S0033291716002804)
Supplement: Supplementary file 1 [file S0033291716002804sup001.docx]

**Supplementary Table S1. Recruitment at pre- and post-intervention (N=2764)***

|  | Pre-intervention - n(%) | Post-intervention – n (%) |
| --- | --- | --- |
| Population interviewed | 1576 (57.02) | 2100 (75.98) |
| Population who migrated out of the village temporarily | 549 (19.86) | 197 (7.13) |
| Population who could not be contacted even after 3 attempts | 583 (21.09) | 401 (14.51) |
| Too ill or dead | 43 (1.56) | 50 (1.81) |
| Refused | 4 (0.001) | 6 (0.002) |
| Incorrect listing of age or duplicate listing | 9 (0.003) | 10 (0.004) |

*N=total adult (≥18 year) population in the village as per household listing

**Supplementary Table S2. Summary for Knowledge, Attitude and Behaviour Questionnaire**

|  | **Question** | **Response** | **Pre-intervention (N=1576) n(%)** | **Post-intervention (N=2100) n(%)** |
| --- | --- | --- | --- | --- |
| **Context*** | Have you seen or heard any information about mental health or mental illness issues in the last year, in any of these ways? (Choose all that apply) | Television | 516(32.74) | 682(32.48) |
|  |  | Other (specify) | 450(28.55) | 428(20.38) |
|  |  | People talking about it | 224(14.21) | 360(17.14) |
|  |  | People talking about it | 148(9.39) | 209(9.95) |
|  |  | Newspaper | 49(3.11) | - |
|  |  | Health centre | - | 89(4.24) |
|  | Where do people in this community first go to seek care for mental illness? | Hospital | 1133(71.89) | 1637(77.95) |
|  |  | Local clinic | 315(19.99) | 285(13.57) |
|  |  | Religious or spiritual advisor | 50(3.17) | 51(2.43) |
|  |  | Traditional healer | 39(2.47) | 45(2.14) |
|  |  | Nowhere/care is not available | 22(1.40) | 34(1.62) |
|  | Do any of the following people you know have a mental illness? | No one known | 1017(64.53) | 1511(71.95) |
|  |  | Neighbour | 258(16.37) | 267(12.71) |
|  |  | Someone else? (specify) | 81(5.14) |  |
|  |  | Family member in this household | 49(3.11) | 69(3.29) |
|  |  | Family member outside this household | 54(3.43) | 70(3.33) |
|  |  | No Neighbour | - | 71(3.38) |
| **Knowledge** | Mentally ill people tend to be violent | Agree strongly | 415(26.33) | 659(31.38) |
|  |  | Agree slightly | 591(37.50) | 770(36.67) |
|  |  | Neither agree nor disagree | 103(6.54) | 137(6.52) |
|  |  | Disagree slightly | 61(3.87) | 119(5.67) |
|  |  | Disagree strongly | 130(8.25) | 201(9.57) |
|  |  | Don’t know | 276(17.51) | 214(10.19) |
|  | People with mental illness cannot live a good, rewarding life. | Agree strongly | 385(24.43) | 1024(48.76) |
|  |  | Agree slightly | 674(42.77) | 701(33.38) |
|  |  | Neither agree nor disagree | 90(5.71) | 93(4.43) |
|  |  | Disagree slightly | 101(6.41) | 88(4.19) |
|  |  | Disagree strongly | 63(4.00) | 74(3.52) |
|  |  | Don’t know | 263(16.69) | 120(5.71) |
|  | People with severe mental health problems can fully recover. | Agree strongly | 670(42.51) | 1065(50.71) |
|  |  | Agree slightly | 565(35.85) | 737(35.10) |
|  |  | Neither agree nor disagree | 72(4.57) | 85(4.05) |
|  |  | Disagree slightly | 61(3.87) | 60(2.86) |
|  |  | Disagree strongly | 33(2.09) | 74(3.52) |
|  |  | Don’t know | 175(11.10) | 79(3.76) |
|  | Medication can be an effective treatment for people with mental health problems. | Agree strongly | 866(54.95) | 1296(61.71) |
|  |  | Agree slightly | 456(28.93) | 580(27.62) |
|  |  | Neither agree nor disagree | 39(2.47) | 55(2.62) |
|  |  | Disagree slightly | 46(2.92) | 48(2.29) |
|  |  | Disagree strongly | 36(2.28) | 55(2.62) |
|  |  | Don’t know | 133(8.44) | 66(3.14) |
| **Attitude** | Mentally ill people shouldn’t get married | Agree strongly | 416(26.40) | 686(32.67) |
|  |  | Agree slightly | 543(34.45) | 553(26.33) |
|  |  | Neither agree nor disagree | 113(7.17) | 192(9.14) |
|  |  | Disagree slightly | 103(6.54) | 239(11.38) |
|  |  | Disagree strongly | 163(10.34) | 280(13.33) |
|  |  | Don’t know | 238(15.10) | 150(7.14) |
|  | People with mental health problems are far less of a danger than most people suppose. | Agree strongly | 454(28.81) | 1071(51.00) |
|  |  | Agree slightly | 644(40.86) | 752(35.81) |
|  |  | Neither agree nor disagree | 108(6.85) | 54(2.57) |
|  |  | Disagree slightly | 83(5.27) | 73(3.48) |
|  |  | Disagree strongly | 53(3.36) | 36(1.71) |
|  |  | Don’t know | 234(14.85) | 114(5.43) |
|  | We need to adopt a far more tolerant attitude toward people with mental illness in our society. | Agree strongly | 970(61.55) | 1671(79.57) |
|  |  | Agree slightly | 391(24.81) | 339(16.14) |
|  |  | Neither agree nor disagree | 46(2.92) | 15(0.71) |
|  |  | Disagree slightly | 43(2.73) | 22(1.05) |
|  |  | Disagree strongly | 18(1.14) | 10(0.48) |
|  |  | Don’t know | 108(6.85) | 43(2.05) |
|  | People with mental health problems should not be given any responsibility. | Agree strongly | 456(28.93) | 1048(49.90) |
|  |  | Agree slightly | 560(35.53) | 562(26.76) |
|  |  | Neither agree nor disagree | 135(8.57) | 125(5.95) |
|  |  | Disagree slightly | 121(7.68) | 154(7.33) |
|  |  | Disagree strongly | 121(7.68) | 125(5.95) |
|  |  | Don’t know | 183(11.61) | 86(4.10) |
| **Behaviour** | If you suffered from a mental health problem would you tell your family or friends | No one | 411(26.08) | 117(5.57) |
|  |  | Friends | 85(5.39) | 86(4.10) |
|  |  | Family | 1080(68.53) | 1897(90.33) |
|  | I would be willing to live with someone with a mental health problem | Agree strongly | 583(36.99) | 1265(60.24) |
|  |  | Agree slightly | 613(38.90) | 604(28.76) |
|  |  | Neither agree nor disagree | 64(4.06) | 22(1.05) |
|  |  | Disagree slightly | 105(6.66) | 76(3.62) |
|  |  | Disagree strongly | 66(4.19) | 102(4.86) |
|  |  | Don’t know | 145(9.20) | 31(1.48) |
|  | I would be willing to work with someone with a mental health problem | Agree strongly | 633(40.16) | 1277(60.81) |
|  |  | Agree slightly | 546(34.64) | 597(28.43) |
|  |  | Neither agree nor disagree | 80(5.08) | 25(1.19) |
|  |  | Disagree slightly | 101(6.41) | 69(3.29) |
|  |  | Disagree strongly | 75(4.76) | 96(4.57) |
|  |  | Don’t know | 141(8.95) | 36(1.71) |
|  | I would be willing to live nearby someone with a mental health problem | Agree strongly | 618(39.21) | 1324(63.05) |
|  |  | Agree slightly | 557(35.34) | 545(25.95) |
|  |  | Neither agree nor disagree | 75(4.76) | 35(1.67) |
|  |  | Disagree slightly | 97(6.15) | 92(4.38) |
|  |  | Disagree strongly | 73(4.63) | 72(3.43) |
|  |  | Don’t know | 156(9.90) | 32(1.52) |
|  | I would be willing to continue a relationship with a friend who developed a mental health problem | Agree strongly | 681(43.21) | 1316(62.67) |
|  |  | Agree slightly | 587(37.25) | 589(28.05) |
|  |  | Neither agree nor disagree | 47(2.98) | 36(1.71) |
|  |  | Disagree slightly | 84(5.33) | 42(2.00) |
|  |  | Disagree strongly | 53(3.36) | 69(3.29) |
|  |  | Don’t know | 124(7.87) | 48(2.29) |

**A Subject can be counted more than once for context questions and only top 5 responses are reported*

**Supplementary Table S3. Summary for Barriers to Access to Care Evaluation – Treatment Stigma Subscale**

| **Question** | **Response** | **Pre-intervention (N=1576),** n(%) | **Post-intervention (N=2100)*,** n(%) |
| --- | --- | --- | --- |
| Concern that I might be seen as weak for having a mental health problem | Not at all | 1106(70.18) | 1548(86.82) |
|  | A little | 355(22.53) | 217(12.17) |
|  | Quite a lot | 87(5.52) | 15(0.84) |
|  | A lot | 28(1.78) | 3(0.17) |
| Concern that it might harm my chances when applying for jobs | Not at all | 192(12.18) | 662(37.13) |
|  | A little | 106(6.73) | 75(4.21) |
|  | Quite a lot | 47(2.98) | 3(0.17) |
|  | A lot | 10(0.63) | 1(0.06) |
|  | NA | 1221(77.47) | 1042(58.44) |
| Concern about what my family might think, say, do or feel | Not at all | 1044(66.24) | 1538(86.26) |
|  | A little | 420(26.65) | 189(10.60) |
|  | Quite a lot | 88(5.58) | 55(3.08) |
|  | A lot | 24(1.52) | 1(0.06) |
| Feeing embarrassed or ashamed | Not at all | 1121(71.13) | 1475(82.73) |
|  | A little | 346(21.95) | 296(16.60) |
|  | Quite a lot | 85(5.39) | 11(0.62) |
|  | A lot | 24(1.52) | 1(0.06) |
| Concern that I might be seen as crazy | Not at all | 1177(74.68) | 1584(88.84) |
|  | A little | 251(15.93) | 179(10.04) |
|  | Quite a lot | 125(7.93) | 17(0.95) |
|  | A lot | 23(1.46) | 3(0.17) |
| Concern that I might be seen as a bad parent | Not at all | 1132(71.83) | 1404(78.74) |
|  | A little | 318(20.18) | 227(12.73) |
|  | Quite a lot | 106(6.73) | 8(0.45) |
|  | A lot | 20(1.27) | 1(0.06) |
|  | NA | 0(0.00) | 143(8.02) |
| Concern that people I know might find out | Not at all | 1076(68.27) | 1559(87.44) |
|  | A little | 382(24.24) | 218(12.23) |
|  | Quite a lot | 97(6.15) | 5(0.28) |
|  | A lot | 21(1.33) | 1(0.06) |
| Concern that people might not take me seriously if they found out I was having professional care | Not at all | 1061(67.32) | 1631(91.48) |
|  | A little | 399(25.32) | 86(4.82) |
|  | Quite a lot | 97(6.15) | 65(3.65) |
|  | A lot | 19(1.21) | 1(0.06) |
| Not wanting a mental health problem to be on my medical records | Not at all | 1280(81.22) | 1698(95.23) |
|  | A little | 168(10.66) | 81(4.54) |
|  | Quite a lot | 54(3.43) | 3(0.17) |
|  | A lot | 74(4.70) | 1(0.06) |
| Concern that my children may be taken into care or that I may lose access or custody without my agreement | Not at all | 1141(72.40) | 1353(75.88) |
|  | A little | 307(19.48) | 272(15.26) |
|  | Quite a lot | 78(4.95) | 7(0.39) |
|  | A lot | 50(3.17) | 2(0.11) |
|  | NA | 0(0.00) | 149(8.36) |
| Concern about what my friends might think, say or do | Not at all | 1042(66.12) | 1545(86.65) |
|  | A little | 398(25.25) | 227(12.73) |
|  | Quite a lot | 110(6.98) | 10(0.56) |
|  | A lot | 26(1.65) | 1(0.06) |
| Concern about what people at work might think, say or do | Not at all | 1036(65.74) | 1479(82.95) |
|  | A little | 386(24.49) | 241(13.52) |
|  | Quite a lot | 120(7.61) | 49(2.75) |
|  | A lot | 34(2.16) | 14(0.79) |

*Though there were 2100 participants at post-intervention, BACE was evaluated on 1783 and all percentages are based on the total of 1783. Items 2, 6, 10 had fewer responses as they had skip options.

**Supplementary Table S4. Mean scores, frequencies and ranks for each barrier in the Barriers to Access to Care Evaluation – Treatment Stigma Subscale (BACE) – Pre-intervention**

| **Question** | **Mean(SD), n** | **%reporting barrier to any degree** | **%reporting as major barrier (a lot)** | **Rank(1=item has highest proportion rating as a major barrier)** |
| --- | --- | --- | --- | --- |
| Concern that I might be seen as weak for having a mental health problem | 0.4 (0.67 ),1576 | 29.82 | 1.78 | 4 |
| Concern that it might harm my chances when applying for jobs | 0.6 (0.81 ),355 | 10.34 | 0.63 | 12 |
| Concern about what my family might think, say, do or feel | 0.4 (0.67 ),1576 | 33.76 | 1.52 | 6 |
| Feeing embarrassed or ashamed | 0.4 (0.66 ),1576 | 28.87 | 1.52 | 6 |
| Concern that I might be seen as crazy | 0.4 (0.69 ),1576 | 25.32 | 1.46 | 8 |
| Concern that I might be seen as a bad parent | 0.4 (0.67 ),1576 | 28.17 | 1.27 | 10 |
| Concern that people I know might find out | 0.4 (0.67 ),1576 | 31.73 | 1.33 | 9 |
| Concern that people might not take me seriously if they found out I was having professional care | 0.4 (0.66 ),1576 | 32.68 | 1.21 | 11 |
| Not wanting a mental health problem to be on my medical records | 0.3 (0.75 ),1576 | 18.78 | 4.70 | 1 |
| Concern that my children may be taken into care or that I may lose access or custody without my agreement | 0.4 (0.73 ),1576 | 27.60 | 3.17 | 2 |
| Concern about what my friends might think, say or do | 0.4 (0.7 ),1576 | 33.88 | 1.65 | 5 |
| Concern about what people at work might think, say or do | 0.5 (0.73 ),1576 | 34.26 | 2.16 | 3 |
| Overall Mean | 0.4 (0.08 ) |  |  |  |

**Supplementary Table S5. Mean scores, frequencies and ranks for each barrier in the Barriers to Access to Care Evaluation – Treatment Stigma Subscale (BACE) - post-intervention**

| **Question** | **Mean(SD), n** | **%reporting barrier to any degree** | **%reporting as major barrier (a lot)** | **Rank(1=item has highest proportion rating as a major barrier)** |
| --- | --- | --- | --- | --- |
| Concern that I might be seen as weak for having a mental health problem | 0.1 (0.39 ),1783 | 11.19 | 0.14 | 2 |
| Concern that it might harm my chances when applying for jobs | 0.1 (0.34 ),741 | 3.76 | 0.05 | 5 |
| Concern about what my family might think, say, do or feel | 0.2 (0.45 ),1783 | 11.67 | 0.05 | 5 |
| Feeing embarrassed or ashamed | 0.2 (0.4 ),1783 | 14.67 | 0.05 | 5 |
| Concern that I might be seen as crazy | 0.1 (0.37 ),1783 | 9.48 | 0.14 | 2 |
| Concern that I might be seen as a bad parent | 0.2 (0.38 ),1640 | 11.24 | 0.05 | 5 |
| Concern that people I know might find out | 0.1 (0.35 ),1783 | 10.67 | 0.05 | 5 |
| Concern that people might not take me seriously if they found out I was having professional care | 0.1 (0.43 ),1783 | 7.24 | 0.05 | 5 |
| Not wanting a mental health problem to be on my medical records | 0.1 (0.23 ),1783 | 4.05 | 0.05 | 5 |
| Concern that my children may be taken into care or that I may lose access or custody without my knowledge | 0.2 (0.4 ),1634 | 13.38 | 0.10 | 4 |
| Concern about what my friends might think, say or do | 0.1 (0.37 ),1783 | 11.33 | 0.05 | 5 |
| Concern about what people at work might think, say or do | 0.2 (0.52 ),1783 | 14.48 | 0.67 | 1 |
| Overall Mean | 0.1 (0.04 ) |  |  |  |
